# Surgical considerations in hyperopic cataract patients: optimizing outcomes and reducing complications

**DOI:** 10.3389/fmed.2025.1624054

**Published:** 2025-11-19

**Authors:** Reem AlHuthail

**Affiliations:** Department of Ophthalmology, College of Medicine, Imam Mohammad Ibn Saud Islamic University, Riyadh, Saudi Arabia

**Keywords:** cataract surgery, hyperopia, anterior chamber depth, phacoemulsification, femtosecond laser-assisted cataract surgery

## Abstract

Cataract surgery in hyperopic individuals presents unique anatomical and physiological challenges, including shallow anterior chambers, increased lens vaults, and a higher risk of intraoperative complications. This review explores key considerations for preoperative assessment, surgical planning, and intraoperative techniques tailored to hyperopic eyes. Preoperative evaluation involves identifying systemic and ocular risk factors, such as alpha-1 blocker use, which may contribute to intraoperative floppy iris syndrome (IFIS). Key assessments include anterior chamber depth (ACD), corneal endothelial integrity, and coexisting conditions like pseudoexfoliation (PXF) syndrome and angle-closure glaucoma. Intraoperative strategies focus on optimizing phacoemulsification parameters, leveraging advanced technologies such as femtosecond laser-assisted cataract surgery (FLACS), and mitigating risks of iris prolapse and endothelial damage. Proper patient positioning, strategic use of viscoelastic agents, and meticulous incision techniques are essential to ensuring safety and surgical success. Postoperative management addresses anisometropia, monitors for complications, and plans for early intervention in the fellow eye to maintain refractive balance. This comprehensive review provides evidence-based guidance to optimize surgical outcomes and minimize complications in hyperopic patients undergoing cataract surgery.

## Introduction

Hyperopia is defined as *a refractive abnormality of the eye in which parallel light rays originating from infinity are focused posterior to the neurosensory retina when accommodation is relaxed* (1).

At birth, humans are mostly hyperopic, and with advancing age, hyperopic eyes develop into emmetropic or even myopic conditions (2, 3). A positive family history significantly influences the development of hyperopia in subsequent generations (4). If neglected following diagnosis, complications including as amblyopia and tropia may arise (5, 6).

## Hyperopia in adults

**Figure 1:**
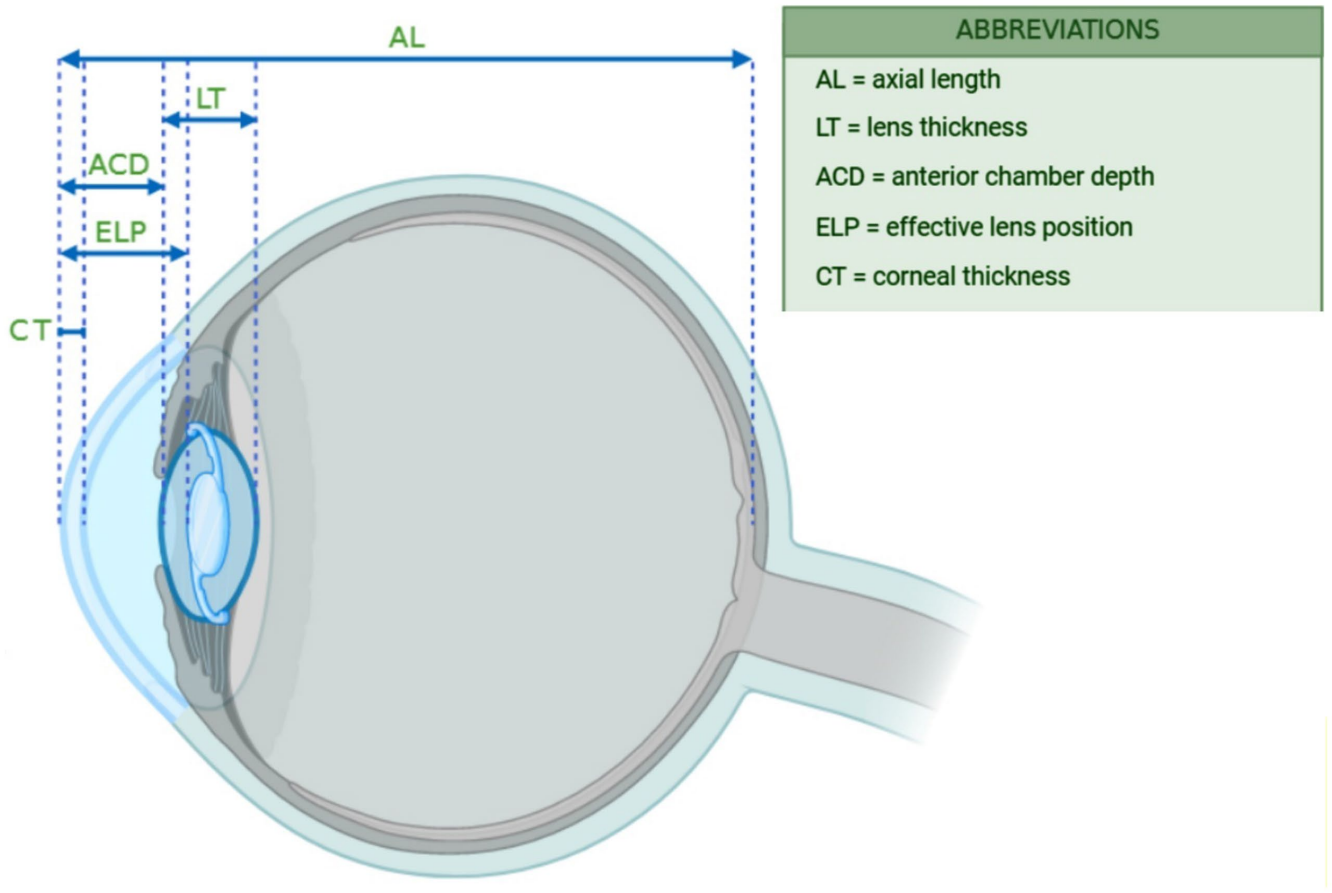
Different ocular parameters in hyperopic eyes.

Hyperopia is a prevalent refractive defect in both juvenile and adult populations, significantly affecting everyday quality of life (7). The global prevalence of hyperopia is estimated at 4.6% in children and 30.9% in adults, with significant variability across different geographic locations (8). Hyperopia is a refractive defect characterized by the focusing of incoming light behind the retina, rather than on it. It is probably influenced by ethnicity, geography, and a familial predisposition to hyperopia or accommodative esotropia, and is classified as low (≤2.00D), moderate (2.00–4.00D), and high (>4.00D). Hyperopic eyes are typically characterized by a short axial length (AL), a relatively thick crystalline lens (LT), and a shallow anterior chamber depth (ACD). The shortened AL shifts the focal point behind the retina, producing hyperopia, while the combination of a large lens within a small globe further reduces ACD. This crowded anterior segment anatomy not only explains the refractive state but also predisposes hyperopic patients to primary angle closure and intraoperative challenges during cataract surgery, particularly with respect to intraocular lens (IOL) power calculation accuracy and anterior chamber stability (9) (Figure 1).

## Etiology of hyperopia

Hyperopia is categorized based on underlying cause into: Axial hyperopia (predominantly simple hyperopia): This is a result from reduction in the anterior–posterior AL of the eyeball. Often influenced by genetic factors. Retinal edema can induce a hyperopic shift. A 1 mm decrease in AL results in 3 diopters of hyperopia (10). Curvature hyperopia: The condition results from the flattening of either the cornea, the lens, or both. An increase of 1 mm in the radius of curvature results in 6 diopters of hyperopia. Index hyperopia: The alteration in the refractive index of the crystalline lens occurs with aging or in individuals with diabetes. The refractive index progressively rises from the center to the periphery. Positional hyperopia, aphakia, or ocular pathological conditions: these conditions arise from the malposition or absence of the crystalline lens (either congenital or acquired) or IOL, resulting in the formation of an aphakic zone inside the refractive medium. Post-traumatic or post-surgical aphakia is a frequent etiology of hyperopia.

To date, no single causal factor for hyperopia has been definitively established. Although relatively uncommon, several genetic variables have been linked to its development. In addition to hereditary and environmental influences, certain acquired conditions contribute to hyperopia, particularly in older adults.

Several recognized conditions associated with hyperopia: cortical cataract (index hyperopia), maternal smoking during gestation, aphakia (congenital or acquired), hyperglycemia, diabetes mellitus, and subsequent management of hyperglycemia in diabetes mellitus: 16p11.2 microdeletion, Myelin regulatory factor gene (MYRF) mutation, and familial predisposition to strabismus. Prolonged space missions can result in retinal and optic nerve head edema, accommodation loss due to total cranial nerve III palsy, internal ophthalmoplegia, or paralysis induced by cycloplegic drops and lorazepam, leading to functional hyperopia. X-linked retinoschisis and senile retinoschisis (11).

## Surgical correction of hyperopia

Laser vision correction and refractive procedures for hyperopia offer a variety of options customized to individual patient requirements and anatomical factors. These include corneal reshaping methods such as Laser-Assisted *In Situ* Keratomileusis (LASIK) and Photorefractive Keratectomy (PRK), As well as lens-based interventions, including Implantable Collamer Lenses (ICL), Refractive Lens Exchange (RLE), or cataract surgery with IOL implantation when cataracts are present. These procedures address varying degrees of hyperopia and associated conditions, such as astigmatism and presbyopia. Each technique has specific indications, benefits, and limitations, requiring a comprehensive preoperative assessment to ensure optimal outcomes. The table summarizes the most common surgical methods for hyperopia correction, highlighting their key characteristics and suitable patient demographics (12–16) (Table 1).

**Table 1:** Management of hyperopic patients.

Surgery name	Description	Indications
Laser-Assisted *In Situ* Keratomileusis (LASIK)	A flap is created on the corneal surface using a microkeratome or femtosecond laser.	Hyperopia up to +6.00 diopters (D) and stable refractive error for at least 1 year.
Photorefractive Keratectomy (PRK)	The corneal epithelium is removed (no flap is created) and the epithelium regenerates over a few days post-surgery.	Hyperopia up to +4.00 D and thin corneas or patients at risk of trauma (e.g., athletes).
Laser Epithelial Keratomileusis (LASEK)	The corneal epithelium is loosened with alcohol and preserved as a flap and the epithelial flap is repositioned post-laser treatment.	Hyperopia up to +4.00 D and patients with thin corneas or dry eyes.
Conductive Keratoplasty (CK)	Radiofrequency energy is applied to the peripheral cornea to shrink collagen fibers, increasing the curvature of the central cornea.	Low hyperopia (+0.75 to +3.00 D) and early presbyopia or patients unsuitable for laser ablation.
Implantable Collamer Lens (ICL)	Specifically designed to treat hyperopia, made of biocompatible Collamer material to minimize risk of rejection, and available in Toric versions to correct hyperopia with astigmatism.	Hyperopia ranging from +3.00 to +20.00 diopters (D), patients with thin corneas or dry eyes, where laser surgeries like LASIK or PRK are unsuitable, stable refractive error for at least one year, and adequate ACD (>2.8 mm).
Refractive Lens Exchange (RLE)	Clear lens replacement with IOL for refractive correction.	Hyperopia (moderate to high) in patients without cataracts, if the patient not fit for laser vision correction procedures or ICL implantation.
Cataract surgery Phacoemulsification with IOL Implantation	Ultrasound-based emulsification of cataracts with IOL implantation.	Cataracts with hyperopia

LASIK, laser-assisted in situ keratomileusis; PRK, photorefractive keratectomy; IOL, intraocular lens; ACD, anterior chamber depth; SMILE, small-incision lenticule extraction; LASEK, laser epithelial keratomileusis; ICL, implantable collamer lens; RLE, refractive lens exchange; CK, conductive keratoplasty.

## Types of IOLs

IOLs are essential components of cataract surgery, designed to replace the eye’s natural lens and restore vision. Technological advancements have led to a wide range of IOL types, each tailored to specific refractive needs and patient preferences. The selection of IOL is depends on factors such as pre-existing refractive error, lifestyle demands, and the desire for spectacle independence.

Selecting the appropriate IOL is crucial for hyperopic patients, who often have high expectations for both near and distance vision correction, to achieve optimal postoperative visual outcomes. Each IOL offers specific advantages that determine its suitability for use while having certain limitations. Therefore, the choice of IOL depends on the ophthalmologist performing the procedure and the patient’s condition, assuming the patient is a suitable candidate. Multifocal IOLs correct vision at various distances, while mono-focal and EDOF IOLs provide vision at a specific distance based on the preoperative plan and may still require spectacle after the procedure (17–28) (Table 2).

**Table 2:** Different types of intraocular lenses used in the cataract surgeries of hyperopic patients.

Lens type	Use	Advantages	Disadvantages
Monofocal IOLs: aiming for Monovision	Correct Dominant eye for distance and the non-dominant eye for near vision.	- Reduce the need for glasses for both near and distance vision. - Cost-effective using monofocal lenses.	- May reduce depth perception. - Require adaptation; not suitable for everyone.
Monofocal IOLs: aiming for Mini monovision	A refined version of monovision where a smaller refractive difference between the two eyes is targeted to balance near and distance vision while preserving better depth perception and reducing adaptation issues.	- Reduces the disparity between the two eyes compared to full monovision. - Easier for the brain to adapt to the smaller difference in focus between the eyes. - Provides functional vision across a broader range of distances without glasses. - Achievable with monofocal IOLs, which are more affordable than premium IOLs.	- May still require glasses for very fine near tasks, such as reading small print. - Not all patients adapt well to even small refractive differences between eyes. - Depth perception is still reduced compared to emmetropic correction in both eyes.
Monofocal IOLs	Single-focus lens to correct hyperopia, typically set for distance vision.	- Affordable and widely available. - Provide clear vision at a single focal distance (near or far).	- Require glasses for other distances (e.g., near tasks).
Toric IOLs	Correct hyperopia and coexisting astigmatism simultaneously.	- Address two refractive errors in one procedure. - Reduce dependence on glasses.	- Require precise alignment during surgery. - Additional cost compared with standard IOLs.
Multifocal IOLs	Correct hyperopia and presbyopia by providing multiple focal points (near, intermediate, and distance).	- Reduce dependence on glasses.	- May cause glare and halos. - Cause reduced contrast sensitivity, especially in low light.
Extended depth of focus (EDOF) IOLs	Correct hyperopia while offering a continuous range of vision (distance to intermediate).	- Cause fewer halos and glare than multifocal IOLs. - Reduce dependence on glasses.	- May still need glasses for fine near tasks. - Not suitable for severe hyperopia.
Accommodative IOLs	Mimic natural lens movement to focus dynamically for various distances.	- Broader range of vision compared with monofocal IOLs. - Reduce dependence on glasses.	- Effectiveness varies based on patient’s ciliary muscle function.

IOL, intraocular lens; EDOF, extended depth of focus.

## Multifocal and EDOF IOLs in hyperopic eyes: indications and contraindications

- Indications:

1. Highly motivated patients prioritizing spectacle independence; realistic about potential halos/glare.
2. Regular corneal optics (low higher-order aberrations), controlled ocular surface disease.
3. Healthy macula/optic nerve and stable IOP.
4. Angle kappa not excessive (centers reasonably with Purkinje reflex), and mesopic pupil compatible with the chosen optic.
5. Low residual astigmatism expectation (treat astigmatism at time of surgery).
6. EDOF/low-add designs or mini-monovision when classic trifocal is borderline.

- Contraindications:

1. Primary angle closure risk with shallow chambers, extensive peripheral anterior synechiae, or unstable IOP.
2. Macular disease, optic neuropathy, or glaucoma with field loss (contrast sensitivity concerns).
3. Irregular cornea (ectasia, scars, high higher-order aberrations), unstable tear film not optimized.
4. Large angle kappa or decentered fixation; small or inconsistent pupils that mis-match the optic’s energy distribution.
5. Unpredictable effective lens position (ELP) in very short eyes or where high-power IOLs are required (defocus curve sensitivity).

If any red flags above are present, EDOF > low-add multifocal > monofocal with mini-monovision is a safer continuum. When targets are borderline, prioritize quality of vision and binocular function over full spectacle independence (29–32).

## Preoperative assessment

### Systemic evaluation before cataract surgery in hyperopia

A comprehensive preoperative systemic assessment is crucial for individuals undergoing cataract surgery, especially those with hyperopia. Hyperopic eyes are structurally more prone to complications, particularly when the anterior chamber is shallow and pseudoexfoliation syndrome (PXF) is present. These eyes are at greater risk of AC collapse during surgery, increasing the likelihood of iris prolapse and capsular rupture (33). Additionally, PXF, which is common in hyperopic eyes, heightens the risk of zonular weakness, poor pupil dilation, and intraoperative complications (34). The use of medications such as alpha-1 blockers for benign prostatic hyperplasia (BPH), which can further increase these risks by contributing to intraoperative floppy iris syndrome (IFIS). Alpha-1 adrenergic receptors are present in the iris dilator muscle. Alpha-1 blockers, such as tamsulosin, alfuzosin, doxazosin, and terazosin, inhibit these receptors, reducing the muscle’s response to vasodilatory agents. This can result in IFIS during surgery, characterized by inadequate preoperative pupil dilation, iris billowing and flaccidity, and iris prolapse into the surgical incisions. Hyperopic eyes are particularly vulnerable to anterior chamber (AC) collapse due to their shallow AC, which exacerbates IFIS. Additionally, hyperopic eyes are at an increased risk of fluctuations in intraocular pressure (IOP), further complicating surgical visualization (35, 36).

### Ocular evaluation

A thorough ocular assessment is essential to identify other risk factors affecting intraoperative and postoperative outcomes. Key components of this assessment include evaluating the patient’s visual potential as Hyperopia is a considerable risk factor for amblyopia in childhood due to the demands of accommodation. Identifying residual amblyopia before surgery is essential for managing postoperative expectations (37). Long-standing hyperopia might result in accommodative esotropia because to severe accommodative strain. Surgeons must consider this to avert postoperative binocular vision complications (38). A detailed anterior segment examination is essential to evaluate the cornea for transparency and curvature, utilizing topography to detect irregular astigmatism, and examining the corneal endothelium helps anticipate the risk of corneal decompensation. Hyperopic eyes with shallow ACD may present borderline conditions that increase this risk (39). Measuring the ACD, degree of pupil dilation, signs of PXF. Inadequate dilation frequently occurs in hyperopic eyes with PXF, elevating zonular tension and intraoperative difficulties (34). Type and grade of cataracts (e.g., nuclear sclerosis, cortical cataract, posterior subcapsular cataract, or polar cataract), with findings correlated to best-corrected vision.

The association between cataract grading and best-corrected visual acuity (BCVA) is conducted to assess cataract severity, hence informing surgical necessity and anticipated results (40).

The lens zonules should be assessed by evaluating pupil dilation, comparing ACD between both eyes, and examining the stability of the lens, which may reveal phacodonesis in cases of severe zonular dialysis.

Measuring intraocular pressure, examining the angle, and evaluating the optic disc is crucial, as hyperopia is a well-known risk factor for angle closure glaucoma because of shallow anterior chamber and relatively thick lens (41). A dilated fundus examination is essential to assess for choroidal folds in cases of acquired hyperopia and signs of posterior microphthalmos in high hyperopia. Additionally, macular edema should be looked for, especially in cases of acquired or induced hyperopia, such as central serous chorioretinopathy (42).

Ultrasound Biomicroscopy (UBM) is particularly valuable in assessing the integrity of the zonular apparatus when clinical signs are inconclusive or in the presence of PXF, as it allows high-resolution visualization of the ciliary body and zonules (43).

## Counseling and planning

### Postoperative anisometropia

Hyperopic eyes are more susceptible to significant refractive discrepancies postoperatively (postoperative anisometropia), especially when the second eye remains untreated. During the interim between procedures, counseling involves informing patients about potential visual impairments, such as dizziness, headaches, or blurred vision. It is also important to emphasize the need for soon-to-be-performed contralateral eye surgery to restore binocular vision and reduce anisometropia (44).

Short-term management may include prescribing contact lenses for the unoperated eye to temporarily balance refractive discrepancies or using high-index spectacle lenses with low thickness to manage anisometropia. Long-term strategies involve planning early cataract surgery in the second eye to synchronize refractive outcomes and restore binocular vision or considering refractive enhancement procedures, such as LASIK or PRK, if indicated (45).

### Ocular biometrics

Ocular biometrics, precisely ACD, and AL are essential for accurately selecting and calculating the appropriate IOL during cataract surgery. The ACD, defined as *the distance between the corneal endothelium and the anterior surface of the lens, is critical for determining the suitability of different IOL types, particularly phakic IOLs*. A shallower ACD may increase the risk of complications related to specific lens types or surgical techniques and can affect IOL power calculations in advanced models. The eye’s AL, measured from the corneal surface to the retina, is crucial for precise IOL power calculation. A reduced AL in hyperopic eyes may lead to overestimating IOL power when traditional methods are used. Conversely, an extended AL in myopic eyes may underestimate IOL power without using contemporary formulas (46, 47). As mentioned earlier, selecting the appropriate IOL type is critical to ensuring optimal surgical outcomes. Precise IOL power estimation is essential in cataract surgery to attain optimal postoperative visual results. The precision of these computations has markedly improved due to technological breakthroughs and the introduction of advanced formulas. Parameters include AL, corneal power (keratometry), AC, and lens thickness are essential for ascertaining the appropriate IOL power. Contemporary models integrate these characteristics, frequently employing machine learning or ray tracing, to accommodate changes in ocular geometry, particularly in individuals with short, long, or post-refractive surgery eyes (46, 47). The selection of the appropriate formula depends on the patient’s ocular characteristics, ensuring customized and accurate outcomes for each individual as shown in Table 3.

**Table 3:** Different calculation formulas used in cataract surgery for IOL power calculations.

Formula	Best for	Features
*SRK/T* (47)	Normal AL (22–26 mm).	Third-generation formula; uses AL, corneal curvature, and AC depth.
*Holladay 1* (48)	Short or normal AL.	Uses AL and corneal power; offers adjustments for surgeon factors.
*Hoffer Q* (87)	Short AL (<22 mm).	Excellent for hyperopic eyes; adjusts for anterior segment size.
*Barrett Universal II* (88)	All AL and refractive states.	Fourth-generation formula with superior accuracy in post-refractive eyes.
*Hill-RBF* (49)	Normal and long AL (>26 mm).	Machine-learning algorithm with no regression assumptions.
*Olsen Formula* (50)	All AL; particularly effective in post-refractive cases.	Uses ray tracing to account for lens position and ocular geometry.
*Haigis Formula* (56)	Eyes with unusual AC depths or post-refractive surgery.	Incorporates AC depth directly into the calculation.
*Panacea Formula* (51)	Post-LASIK/PRK eyes with altered corneal power.	Designed for corneas with altered biomechanics or previous refractive surgery.

AL, axial length; LASIK, laser-assisted in situ keratomileusis; PRK, photorefractive keratectomy; AC, anterior chamber.

In hyperopic patients with short AL, studies suggest that the *Barrett Universal II* and *Holladay 2* formulas offer superior accuracy over older third-generation formulas like *SRK/T* and *Hoffer Q*. The *Kane formula*, which incorporates artificial intelligence-driven optimization, also demonstrates high predictive accuracy across various eye lengths, particularly in post-refractive and short eyes. The *Haigis formula*, which directly uses ACD values, performs well in eyes with unusual anterior chamber dimensions but may be less accurate than *Barrett* or *Kane* in high hyperopia (48–50).

### Hyperopic patients who previously underwent refractive surgery

In hyperopic patients who have undergone prior corneal refractive surgery (e.g., LASIK or PRK), cataract surgery presents unique challenges in both biometric assessment and IOL power calculation. These eyes often exhibit altered anterior corneal curvature, loss of the normal anterior–posterior corneal power relationship, and disrupted ELP prediction, all of which can compromise the accuracy of standard IOL formulas. In particular, traditional keratometry tends to overestimate corneal power in post-hyperopic LASIK/PRK eyes, increasing the risk of postoperative hyperopic refractive surprises (51–53). These inaccuracies are exacerbated in the absence of historical refractive data, which is often the case. To address these issues, surgeons should employ advanced technologies such as ray-tracing biometers or swept-source OCT devices, which more accurately measure total corneal power (54, 55). The use of dedicated post-refractive surgery formulas is essential in these cases; among the most validated options are the *Barrett True-K* (no-history), *Haigis-L, Shammas-PL*, and the artificial intelligence-driven *Kane* post-refractive formula, all of which demonstrate improved accuracy over traditional third-generation formulas. Intraoperative aberrometry may also be considered for real-time confirmation of IOL power. With respect to IOL selection, multifocal lenses are generally discouraged in these eyes due to potential corneal irregularity and reduced contrast sensitivity. Instead, monofocal or enhanced monofocal IOLs are preferred, while EDOF lenses may be cautiously considered in patients with regular topography and stable corneal surfaces. Incorporating these adjustments into preoperative planning helps mitigate refractive surprises and optimize visual outcomes in this high-risk subgroup (56–59).

## Type of anesthesia

Cataract surgery can be performed using various anesthesia methods, including local (regional) anesthesia such as retrobulbar, peribulbar, sub-Tenon’s injection, intracameral, and topical anesthesia and, in rare cases, general anesthesia. The chosen anesthesia method should be communicated to the patient to ensure awareness of expected pain, discomfort, consciousness level, visual experiences, and potential risks. Studies evaluating cataract surgery outcomes measured by visual acuity, visual function, complications, adverse medical events, and patient satisfaction have not shown significant differences between anesthesia techniques. Intravenous (IV) sedation is often used to supplement local anesthesia, enhancing patient comfort and cooperation during surgery. Needle- or blunt-cannula-based anesthesia techniques may pose challenges not encountered with topical or intracameral methods. Potential complications include strabismus, globe perforation, retrobulbar hemorrhage, intravascular or subarachnoid injection, and macular infarction. Patients with posterior staphylomas or scleral buckles are at an increased risk of globe perforation with peribulbar or retrobulbar injections (60).

## Surgical considerations

### Positioning of the patient

Optimal patient positioning during cataract surgery is crucial, particularly for hyperopic individuals who may present anatomical challenges. Hyperopic eyes often have a shallow anterior chamber, and deep-set eyes can further complicate surgical access and visualization. Proper alignment helps mitigate these difficulties, ensuring a safer and more efficient procedure.

### Positioning techniques

Head position: The angle of the headrest is adjusted by placing the patient’s head in modest extension (chin lifted) to enhance the surgeon’s approach angle and improve visualization of the anterior area. Aligning the visual axis is performed to ensure the patient’s visual axis coincides with the microscope’s center, hence minimizing parallax errors (61).

Table position: Modifying the operating table’s height to enable the surgeon to sustain an ergonomic posture while ensuring stability in the surgical field. Adjusting the table to a Trendelenburg position can, in certain instances, facilitate the deepening of the AC by utilizing gravity to displace intraocular contents posteriorly (62).

Utilization of Eye Speculum: Choosing a suitable speculum for patients with deep-set eyes to guarantee complete visibility of the surgical area while avoiding excessive strain on the globe (63).

A secondary device (e.g., chopper or cyclodialysis spatula) is employed to stabilize the globe or retract tissue, which may be essential for enhanced control (64).

### Decreasing vitreous pressure

1. Honan Intraocular Pressure Reducer: The Honan balloon is a device applied externally to the eye to provide gentle compression, thereby reducing IOP before surgery (65).
2. Preoperative Intravenous Mannitol: Mannitol is an osmotic diuretic administered intravenously to decrease IOP by drawing fluid out of the vitreous body (66).
3. Limited Anterior Vitrectomy: This surgical procedure involves removing a portion of the anterior vitreous to alleviate vitreous pressure, particularly in cases where vitreous prolapse occurs during cataract surgery or other anterior segment procedures (67).

Hyperopic eyes with a shallower anterior chamber and positive posterior pressure may respond less favorably to peribulbar or retrobulbar anesthesia due to increased risk of globe compression and shallow chamber collapse. Unlike emmetropic or myopic eyes, hyperopic eyes often have crowded anterior segments and a deeper orbital set, which necessitates more strategic positioning and greater care in globe stabilization during surgery.

## Clear corneal incision

The corneal incision is typically made in the temporal or superior region. These small, self-sealing incisions, ranging from 2.2 to 2.8 mm, are characteristic of modern phacoemulsification techniques. The incision is trapezoidal, with a narrower internal width and a broader external breadth to enhance stability and self-sealing properties. To optimize surgical access, maintain anterior chamber stability, and reduce induced astigmatism, temporal clear corneal incisions are modified to be more anterior and elongated. This adjustment helps minimize intraoperative fluctuations, decrease the risk of iris prolapse, and improve overall wound integrity (68).

A keratome blade, typically measuring 2.2–2.8 mm, is then used to create a precise, tunneled incision by penetrating the cornea at an angle of 30–45 degrees. To enhance anterior chamber stability and minimize iris prolapse, the incision is positioned more anteriorly and extended in length. This modification helps maintain chamber depth and improves wound integrity. Postoperatively, the wound edges are properly hydrated to ensure a watertight seal and promote rapid healing (69).

### Viscoelastic agents

They play a crucial role in maintaining anterior chamber stability during surgery. Dispersive viscoelastics provide corneal endothelium protection, while cohesive viscoelastics, such as sodium hyaluronate 1.4%, enhance chamber volume and deepen the anterior chamber. To maintain intraoperative stability, wound margin hydration is performed to achieve sufficient stromal hydration, preserving anterior chamber integrity (70).

## Capsulorrhexis in shallow anterior chambers

### Capsulorrhexis in shallow anterior chambers

Performing a well-centered and intact capsulorrhexis is a crucial step in cataract surgery. In hyperopic eyes with shallow anterior chambers, the risk of peripheral tearing increases due to limited working space, elevated intraocular pressure (IOP), and increased zonular stress. Specific techniques and instruments can mitigate these risks and improve surgical outcomes (61).

### Techniques for safe capsulorrhexis

1. Utilization of Ophthalmic Viscosurgical Devices (OVDs):

Maintaining a stable ACD is critical during capsulorrhexis. Regularly refilling the chamber with cohesive OVDs prevents anterior capsule collapse. The soft-shell technique combines a dispersive OVD, such as *Viscoat*, to protect the endothelium, with a cohesive OVD, like *ProVisc*, to stabilize the anterior chamber and facilitate controlled capsulorrhexis (71).
Bimanual procedures involve use both hands to manipulate equipment, hence enhancing control during the development of capsulorrhexis in congested anterior segments (72).

The use of capsule staining agents, such as trypan blue dye, improves the visibility of the anterior capsule in eyes with a diminished red reflex, facilitating safer tear management (73). Additionally, microincision devices, such as fine forceps or cystotomes, enhance maneuverability in confined spaces, reducing the likelihood of peripheral tears (74).
Femtosecond Laser-Enhanced Capsulotomy:

The femtosecond laser creates a precise and uniform capsulorrhexis, which is particularly beneficial when the ACD is less than 2–2.5 mm. This approach reduces the risk of radial tears, improves centration and uniformity of the capsulotomy, and alleviates zonular stress, especially in cases of PXF (75).

*CAPSULaser*:

*CAPSULaser* (*EXCEL-LENS, Inc., Livermore, CA*) is a Class 4 solid-state laser operating at a wavelength of 590 ± 3 nm with continuous wave output at 100% capacity. The device can be effortlessly affixed to the surgical microscope and features independent pedal control. The laser was required to undergo a standardized protocol prior to the operative day. Each technique was validated through the utilization of a software key. The diameter of the CCC can be adjusted from 4.0 to 5.5 mm in 0.1 mm increments. The method of action entails the specific absorption of the dye by the trypan blue anterior capsule (76).

## Phacoemulsification parameter settings in cataract surgery

Optimizing phacoemulsification parameters is crucial for ensuring safety and efficacy, especially in hyperopic eyes with a shallow AC. Adjusting surgical settings can protect the corneal endothelium and reduce complications such as iris prolapse.

A well-controlled, centered capsulorhexis is essential for safe lens removal in a shallow AC. Using smaller phaco tips (2.2–2.8 mm) minimizes congestion, while reduced infusion and aspiration rates help maintain chamber stability by decreasing turbulence. If necessary, a paracentesis incision allows for supplemental infusion or viscoelastic injection, further enhancing intraoperative control (71).

Phacoemulsification is performed at low-flow settings to preserve ACD, utilizing either a divide-and-conquer or stop-and-chop technique to minimize zonular stress. In cases where phacoemulsification poses a high risk of endothelial injury due to anterior segment congestion, Manual Small-Incision Cataract Surgery (MSICS) may be a safer alternative. Additionally, FLACS enables capsulorhexis and lens fragmentation before ocular entry, reducing surgical manipulation in a shallow AC (72, 75).

### Combined procedures in primary angle closure/primary angle closure glaucoma: phaco-goniosynechialysis and phaco-trabeculotomy

In primary angle closure/primary angle closure glaucoma, lens removal deepens the anterior chamber and widens the angle, but long-standing peripheral anterior synechiae can limit trabecular access. Combining phaco with goniosynechialysis (GSL) can mechanically strip peripheral anterior synechiae and restore outflow. Where angle tissue is accessible, trabeculotomy (e.g., ab interno techniques) may further reduce IOP and medication burden.

#### Indications

- Significant PAS (>180°) or gonioscopic evidence of non-opening angles after indentation.
- Inadequate IOP control or medication intolerance despite laser peripheral iridotomy/medical therapy.
- Evidence of progression (retinal nerve fiber layer change/field loss) where angle rehabilitation may help.

#### Procedure selection

- Phaco and GSL: preferred when peripheral anterior synechiae is the primary issue and angle landmarks are visible after lens extraction and viscodilation.
- Phaco and trabeculotomy: consider if trabecular outflow is clearly limited and Schlemm’s canal access is feasible; in primary angle closure eyes, success depends on peripheral anterior synechiae extent and trabecular meshwork health.

In appropriately selected primary angle closure/primary angle closure glaucoma eyes, phaco alone often lowers IOP and meds; adding GSL and/or trabeculotomy may yield additional IOP reduction and medication sparing, especially with extensive peripheral anterior synechiae (77–80).

### Protecting the corneal endothelium

Hyperopic eyes with a shallow AC are at increased risk of corneal endothelial injury due to restricted working space and the close proximity of the phaco tip to the corneal endothelium. The following parameter configurations can help mitigate endothelial damage:

- Phaco Power Modulation: Torsional or longitudinal phacoemulsification reduces heat production and turbulence compared to purely longitudinal phaco. Utilizing low-power settings with burst or pulse modes minimizes unnecessary energy delivery. Recommended settings include 30–40% phaco power with a 50% duty cycle. Vacuum levels should be maintained at 200–300 mmHg, with an aspiration flow rate of 20–25 mL/min, ensuring controlled vacuum and aspiration rates (81, 82).
- Use of Viscoelastics: Dispersive viscoelastics (e.g., Viscoat) should be used to coat and protect the endothelium throughout the procedure, with frequent replenishment to maintain a barrier between the phaco tip and the cornea.
- Irrigation Solutions and Fluid Management: Balanced salt solution (BSS) with additives such as glutathione or bicarbonate helps preserve corneal hydration and endothelial cell function. Lowering the infusion bottle height regulates inflow pressure and prevents fluctuations in the AC.

### Reducing the risk of iris prolapse

Iris prolapse frequently occurs in shallow anterior chambers due to elevated intraocular pressure (IOP) or improper incision formation. The following techniques help mitigate this risk:

Incision construction: Create a properly sized, self-sealing clear corneal incision (2.2–2.8 mm) with an adequate tunnel length (1.5–2 mm). Avoid excessively anterior or posterior placement, as improper positioning increases the risk of prolapse.
Fluidic adjustments: Maintain bottle height within a reasonable range (e.g., 70–90 cm) to prevent sudden IOP spikes that may push the iris outward. Ensure anterior chamber stability by using moderate vacuum settings (200–250 mmHg) and aspiration flow rates (20 mL/min) to prevent chamber collapse.
Use of ophthalmic viscosurgical devices (OVDs): Inject cohesive OVDs to deepen the anterior chamber and stabilize the iris.
Management of intraoperative floppy iris syndrome (IFIS): In patients on alpha-1 blockers, employ iris hooks or a Malyugin ring to prevent prolapse.
Irrigation sleeve positioning: Ensure the sleeve directs fluid flow away from the iris to minimize turbulence and prolapse risk.
Advanced fluidic technologies: Utilize modern systems, such as active fluidics, to maintain stable IOP and prevent sudden fluctuations (35, 83).

## Patient-reported outcomes in hyperopic cataract surgery

- VF-14 (Visual Function Index-14). Measures task-based visual function across 14 activities; scored 0–100 (higher = better). It is responsive to cataract surgery and captures everyday limitations (driving, reading). In hyperopic eyes—where depth of focus strategies and lens design can affect functional vision—VF-14 can contextualize objective refractive outcomes with real-world impact (84).
- Catquest-9SF. A Rasch-scaled, 9-item questionnaire widely used for routine cataract outcome assessment. It is sensitive to quality-of-vision improvements and can detect differences between monofocal vs. EDOF/multifocal strategies relevant to hyperopes targeting spectacle independence (85).
- National Eye Institute–Refractive Error Quality of Life (NEI-RQL-42). A 42-item instrument focused on refractive quality of life (clarity, expectation, dependence on correction, symptoms). Its multidomain structure makes it useful for evaluating dysphotopsia/contrast trade-offs in multifocal or EDOF implantation (86).

## Postoperative management

Immediate postoperative care encompasses medications such as topical antibiotics, including moxifloxacin, prescribed to avert infections; corticosteroids, such as prednisolone acetate, utilized to control inflammation; and nonsteroidal anti-inflammatory drugs (NSAIDs), like ketorolac, which may be administered to relieve pain and diminish the risk of cystoid macular edema (CME). Initial evaluation of wound integrity, IOP, IOL placement, and early sequelae include corneal edema or endophthalmitis on first day. Subsequently, after the initial week, assess for ongoing healing and the removal of inflammation. A thorough evaluation to determine visual outcomes, remaining refractive error, and retinal health following the initial month.

## Recommendations

In conclusion, we recommend the following approach:

Systemic and ocular assessment: Conduct a thorough review of the patient’s medical history, including medications (e.g., alpha-1 blockers) that may contribute to intraoperative floppy iris syndrome (IFIS). Assess ACD, corneal endothelial cell density, and AL to identify hyperopia-related risks. Additionally, evaluate for coexisting conditions, angle-closure glaucoma, PXF syndrome, and amblyopia.
Operative planning: Incorporate advanced imaging and diagnostic tools, such as biometry and corneal topography, to inform surgical strategy. Simulate postoperative visual outcomes to align patient expectations, particularly in cases of amblyopia or anisometropia. Plan for early intervention in the fellow eye to minimize the risk of postoperative anisometropia.
Intraoperative management: Optimize phacoemulsification parameters to reduce endothelial injury and stabilize the anterior chamber. Use low-flow fluidics and dispersive viscoelastics to protect ocular structures. Consider FLACS to enhance precision and reduce zonular stress, particularly in shallow anterior chambers. Ensure the patient is positioned meticulously, with adjustments to the headrest and operating table, to improve visualization and minimize surgical complexity.

## References

[1] McCullough SJ Doyle L Saunders KJ. Intra- and inter- examiner repeatability of cycloplegic retinoscopy among young children. Ophthalmic Physiol Opt. (2017) 37:16–23. doi: 10.1111/opo.12341, PMID: 28030881

[2] Semeraro F Forbice E Nascimbeni G Cillino S Bonfiglio VME Filippelli ME . Ocular refraction at birth and its development during the first year of life in a large cohort of babies in a single Center in Northern Italy. Front Pediatr. (2019) 7:539. doi: 10.3389/fped.2019.00539, PMID: 32083036 PMC7001530

[3] Yahya AN Sharanjeet-Kaur S Akhir SM. Distribution of refractive errors among healthy infants and young children between the age of 6 to 36 months in Kuala Lumpur, Malaysia-a pilot study. Int J Environ Res Public Health. (2019) 16:4730. doi: 10.3390/ijerph16234730, PMID: 31783494 PMC6926593

[4] Tarczy-Hornoch K. The epidemiology of early childhood hyperopia. Optom Vis Sci. (2007) 84:115–23. doi: 10.1097/OPX.0b013e318031b674, PMID: 17299341

[5] Lin PW Chang HW Lai IC Teng MC. Visual outcomes after spectacles treatment in children with bilateral high refractive amblyopia. Clin Exp Optom. (2016) 99:550–4. doi: 10.1111/cxo.12412, PMID: 27426739

[6] Laiginhas R Figueiredo L Rothwell R Geraldes R Chibante J Ferreira CC. Long-term refractive outcomes in children with early diagnosis of moderate to high hyperopia. Strabismus. (2020) 28:61–6. doi: 10.1080/09273972.2020.1752265, PMID: 32316817

[7] Kandel H Khadka J Goggin M Pesudovs K. Impact of refractive error on quality of life: a qualitative study. Clin Experiment Ophthalmol. (2017) 45:677–88. doi: 10.1111/ceo.12954, PMID: 28370795

[8] Hashemi H Fotouhi A Yekta A Pakzad R Ostadimoghaddam H Khabazkhoob M. Global and regional estimates of prevalence of refractive errors: systematic review and meta-analysis. J Curr Ophthalmol. (2018) 30:3–22. doi: 10.1016/j.joco.2017.08.009, PMID: 29564404 PMC5859285

[9] Marinescu MC Dascalescu DM Constantin MM Coviltir V Potop V Stanila D . Particular anatomy of the hyperopic eye and potential clinical implications. Medicina (Kaunas). (2023) 59:1660. doi: 10.3390/medicina59091660, PMID: 37763779 PMC10536421

[10] Strang NC Schmid KL Carney LG. Hyperopia is predominantly axial in nature. Curr Eye Res. (1998) 17:380–3. doi: 10.1080/02713689808951218, PMID: 9561829

[11] Harb EN Wildsoet CF. Origins of refractive errors: environmental and genetic factors. Annu Rev Vis Sci. (2019) 5:47–72. doi: 10.1146/annurev-vision-091718-015027, PMID: 31525141 PMC11827892

[12] Chi Q Yang T Chen Y. A systematic review and meta-analysis on intraocular lens implantation with different performances for the treatment of cataract. Ann Palliat Med. (2022) 11:260–71. doi: 10.21037/apm-21-3767, PMID: 35144417

[13] Day AC Burr JM Bennett K Bunce C Doré CJ Rubin GS . Femtosecond laser-assisted cataract surgery versus phacoemulsification cataract surgery (FACT): a randomized noninferiority trial. Ophthalmology. (2020) 127:1012–9. doi: 10.1016/j.ophtha.2020.02.028, PMID: 32386810 PMC7397499

[14] Wang H Chen X Xu J Yao K. Comparison of femtosecond laser-assisted cataract surgery and conventional phacoemulsification on corneal impact: a meta-analysis and systematic review. PLoS One. (2023) 18:e0284181. doi: 10.1371/journal.pone.0284181, PMID: 37058458 PMC10104330

[15] Alio JL Grzybowski A El Aswad A Romaniuk D. Refractive lens exchange. Surv Ophthalmol. (2014) 59:579–98. doi: 10.1016/j.survophthal.2014.04.004, PMID: 25127929

[16] Snellingen T Evans JR Ravilla T Foster A. Surgical interventions for age-related cataract. Cochrane Database Syst Rev. (2002) 2:Cd001323. doi: 10.1002/14651858.CD001323 12076405

[17] Authors HC Bailey S CADTH Health Technology Review. Intraocular lenses for cataract surgery: CADTH health technology review. Ottawa (ON): Canadian Agency for Drugs and Technologies in Health (2023). 38320059

[18] Davis G. The evolution of cataract surgery. Mo Med. (2016) 113:58–62. PMID: 27039493 PMC6139750

[19] Nam JW Lee JH Zhang H Sung MS Park SW. Comparison of the visual outcomes of enhanced and standard Monofocal intraocular Lens implantations in eyes with early Glaucoma. J Clin Med. (2023) 12:5830. doi: 10.3390/jcm12185830, PMID: 37762769 PMC10531790

[20] Shah S Peris-Martinez C Reinhard T Vinciguerra P. Visual outcomes after cataract surgery: multifocal versus monofocal intraocular lenses. J Refract Surg. (2015) 31:658–66. doi: 10.3928/1081597X-20150611-01, PMID: 26465253

[21] Knorz MC. Multifocal intraocular lenses: overview of their capabilities, limitations, and clinical benefits. J Refract Surg. (2008) 24:215–7. doi: 10.3928/1081597X-20080301-01, PMID: 18416254

[22] Cochener B. Prospective clinical comparison of patient outcomes following implantation of trifocal or bifocal intraocular lenses. J Refract Surg. (2016) 32:146–51. doi: 10.3928/1081597X-20160114-01, PMID: 27027620

[23] Kanclerz P Toto F Grzybowski A Alio JL. Extended depth-of-field intraocular lenses: an update. Asia Pac J Ophthalmol (Phila). (2020) 9:194–202. doi: 10.1097/APO.0000000000000296, PMID: 32511121 PMC7299221

[24] Piovella M Colonval S Kapp A Reiter J Van Cauwenberge F Alfonso J. Patient outcomes following implantation with a trifocal toric IOL: twelve-month prospective multicentre study. Eye (Lond). (2019) 33:144–53. doi: 10.1038/s41433-018-0076-5, PMID: 30190549 PMC6328597

[25] Sundy M McKnight D Eck C Rieger F 3rd. Visual acuity outcomes of Toric Lens implantation in patients undergoing cataract surgery at a residency training program. Mo Med. (2016) 113:40–3. PMID: 27039489 PMC6139744

[26] Ong HS Evans JR Allan BD. Accommodative intraocular lens versus standard monofocal intraocular lens implantation in cataract surgery. Cochrane Database Syst Rev. (2014) 2014:Cd009667. doi: 10.1002/14651858.CD009667.pub2, PMID: 24788900 PMC10505746

[27] Sandoval HP Potvin R Solomon KD. Comparing visual performance and subjective outcomes with an enhanced Monofocal intraocular Lens when targeted for Emmetropia or Monovision. Clin Ophthalmol. (2023) 17:3693–702. doi: 10.2147/OPTH.S442752, PMID: 38058694 PMC10697088

[28] Wróbel-Dudzińska D Moura-Coelho N Palma-Carvajal F Zebdeh A Manero F Güell JL. Ten-year outcomes of pseudophakic mini-monovision correction of hyperopic presbyopia. J Cataract Refract Surg. (2023) 49:367–72. doi: 10.1097/j.jcrs.0000000000001116, PMID: 36729769

[29] Liu H Li FF Xia HJ Zhou J. Visual quality after implantation of trifocal intraocular lenses in highly myopic eyes with different axial lengths. Int J Ophthalmol. (2021) 14:371–7. doi: 10.18240/ijo.2021.03.06, PMID: 33747811 PMC7930543

[30] de Vries NE Nuijts RM. Multifocal intraocular lenses in cataract surgery: literature review of benefits and side effects. J Cataract Refract Surg. (2013) 39:268–78. doi: 10.1016/j.jcrs.2012.12.002, PMID: 23332253

[31] Breyer DRH Kaymak H Ax T Kretz FTA Auffarth GU Hagen PR. Multifocal intraocular lenses and extended depth of focus intraocular lenses. Asia Pac J Ophthalmol (Phila). (2017) 6:339–49. doi: 10.22608/APO.2017186, PMID: 28780781

[32] Kohnen T Suryakumar R. Extended depth-of-focus technology in intraocular lenses. J Cataract Refract Surg. (2020) 46:298–304. doi: 10.1097/j.jcrs.0000000000000109, PMID: 32126045

[33] Jonas JB Nangia V Gupta R Khare A Sinha A Agarwal S . Anterior chamber depth and its associations with ocular and general parameters in adults. Clin Experiment Ophthalmol. (2012) 40:550–6. doi: 10.1111/j.1442-9071.2011.02748.x, PMID: 22171546

[34] Sangal N Chen TC. Cataract surgery in pseudoexfoliation syndrome. Semin Ophthalmol. (2014) 29:403–8. doi: 10.3109/08820538.2014.959189, PMID: 25325866

[35] Chang DF Campbell JR. Intraoperative floppy iris syndrome associated with tamsulosin. J Cataract Refract Surg. (2005) 31:664–73. doi: 10.1016/j.jcrs.2005.02.027, PMID: 15899440

[36] Neff KD Sandoval HP Fernández de Castro LE Nowacki AS Vroman DT Solomon KD. Factors associated with intraoperative floppy iris syndrome. Ophthalmology. (2009) 116:658–63. doi: 10.1016/j.ophtha.2008.12.026, PMID: 19243826

[37] Sprunger DT Lambert SR Hercinovic A Morse CL Repka MX Hutchinson AK . Esotropia and exotropia preferred practice pattern®. Ophthalmology. (2023) 130:P179–p221. doi: 10.1016/j.ophtha.2022.11.002, PMID: 36526451 PMC10655158

[38] Birch EE Fawcett SL Morale SE Weakley DR Jr Wheaton DH. Risk factors for accommodative Esotropia among Hypermetropic children. Invest Ophthalmol Vis Sci. (2005) 46:526–9. doi: 10.1167/iovs.04-0618, PMID: 15671278

[39] Ianchulev T Lane S Masis M Lass JH Benetz BA Menegay HJ . Corneal endothelial cell density and morphology after phacoemulsification in patients with primary open-angle Glaucoma and cataracts: 2-year results of a randomized multicenter trial. Cornea. (2019) 38:325–31. doi: 10.1097/ICO.0000000000001826, PMID: 30614901

[40] Bourdon H Trinh L Robin M Baudouin C. Assessing the correlation between swept-source optical coherence tomography lens density pattern analysis and best-corrected visual acuity in patients with cataracts. BMJ Open Ophthalmol. (2021) 6:e000730. doi: 10.1136/bmjophth-2021-000730, PMID: 34046526 PMC8126301

[41] Zhou S Pardeshi AA Burkemper B Apolo G Cho A Jiang X . Refractive error and anterior chamber depth as risk factors in primary angle closure disease: the Chinese American eye study. J Glaucoma. (2023) 32:257–64. doi: 10.1097/IJG.0000000000002154, PMID: 36847699 PMC10065888

[42] Coan LJ Williams BM Krishna Adithya V Upadhyaya S Alkafri A Czanner S . Automatic detection of glaucoma via fundus imaging and artificial intelligence: a review. Surv Ophthalmol. (2023) 68:17–41. doi: 10.1016/j.survophthal.2022.08.005, PMID: 35985360

[43] He M Wang D Jiang Y. Overview of ultrasound biomicroscopy. J Curr Glaucoma Pract. (2012) 6:25–53. doi: 10.5005/jp-journals-10008-1105, PMID: 27990069 PMC5159457

[44] Langenbucher A Szentmáry N. Anisometropia and aniseikonia--unsolved problems of cataract surgery. Klin Monatsbl Augenheilkd. (2008) 225:763–9. doi: 10.1055/s-2008-1027601, PMID: 18759207

[45] Nogaj S Dubas K Michalski A Stopa M. Spectacle correction of anisometropia following cataract surgery. Klinika Oczna/Acta Ophthalmologica Polonica. (2021) 123:185–8.

[46] Kim JW Eom Y Yoon EG Choi Y Song JS Jeong JW . Algorithmic intraocular lens power calculation formula selection by keratometry, anterior chamber depth and axial length. Acta Ophthalmol. (2022) 100:e701–9. doi: 10.1111/aos.14956, PMID: 34378871 PMC9292369

[47] Sedaghat MR Azimi A Arasteh P Tehranian N Bamdad S. The relationship between anterior chamber depth, axial length and intraocular Lens power among candidates for cataract surgery. Electron Physician. (2016) 8:3127–31. doi: 10.19082/3127, PMID: 27957314 PMC5133039

[48] Cooke DL Aramberri J. Holladay formulas. In: Intraocular lens calculations: Springer (2024). 661–72.

[49] Hill WE Haehnle J. Hill-RBF: improving IOL power selection by artificial intelligence. In: Aramberri J Hoffer KJ Olsen T Savini G Shammas HJ, editors. Intraocular lens calculations. Cham: Springer International Publishing (2024). 637–48.

[50] Stopyra W Langenbucher A Grzybowski A. Intraocular Lens power calculation formulas-a systematic review. Ophthalmol Ther. (2023) 12:2881–902. doi: 10.1007/s40123-023-00799-6, PMID: 37698825 PMC10640516

[51] Savini G Hoffer KJ Balducci N Barboni P Schiano-Lomoriello D. Comparison of formula accuracy for intraocular lens power calculation based on measurements by a swept-source optical coherence tomography optical biometer. J Cataract Refract Surg. (2020) 46:27–33. doi: 10.1016/j.jcrs.2019.08.044, PMID: 32050229

[52] Feiz V. Intraocular lens power calculation after corneal refractive surgery. Middle East Afr J Ophthalmol. (2010) 17:63–8. doi: 10.4103/0974-9233.61219, PMID: 20543939 PMC2880376

[53] Li L Yuan L Yang K Wu Y Hua X Wang Y . Comparative analysis of IOL power calculations in postoperative refractive surgery patients: a theoretical surgical model for FS-LASIK and SMILE procedures. BMC Ophthalmol. (2023) 23:416. doi: 10.1186/s12886-023-03164-0, PMID: 37845633 PMC10578000

[54] Li H Nan L Li J Song H. Accuracy of intraocular lens power calculation formulae after laser refractive surgery in myopic eyes: a meta-analysis. Eye Vis (Lond). (2020) 7:37. doi: 10.1186/s40662-020-00188-1 32656291 PMC7339492

[55] Budiman B Knoch AMH Boesoirie SF Budiman NK Irfani I Sugiarti ED . Agreement between IOLMaster 700 and Pentacam AXL for IOL power measurement in patients with high myopia. Indian J Ophthalmol. (2024) 72:1021–5. doi: 10.4103/IJO.IJO_1350_23, PMID: 38905462 PMC11329815

[56] Haigis W. Intraocular lens calculation after refractive surgery for myopia: Haigis-L formula. J Cataract Refract Surg. (2008) 34:1658–63. doi: 10.1016/j.jcrs.2008.06.029, PMID: 18812114

[57] Gasparian SA Nassiri S You H Vercio A Hwang FS. Intraoperative aberrometry compared to preoperative Barrett true-K formula for intraocular lens power selection in eyes with prior refractive surgery. Sci Rep. (2022) 12:7357. doi: 10.1038/s41598-022-11462-8, PMID: 35513494 PMC9072433

[58] Shammas HJ Shammas MC. No-history method of intraocular lens power calculation for cataract surgery after myopic laser in situ keratomileusis. J Cataract Refract Surg. (2007) 33:31–6. doi: 10.1016/j.jcrs.2006.08.045, PMID: 17189790

[59] Kane JX Van Heerden A Atik A Petsoglou C. Intraocular lens power formula accuracy: comparison of 7 formulas. J Cataract Refract Surg. (2016) 42:1490–500. doi: 10.1016/j.jcrs.2016.07.021, PMID: 27839605

[60] Olson RJ Braga-Mele R Chen SH Miller KM Pineda R Tweeten JP . Cataract in the adult eye preferred practice pattern®. Ophthalmology. (2017) 124:P1–P119. doi: 10.1016/j.ophtha.2016.09.027, PMID: 27745902

[61] Isawumi MA Ademola-Popoola D. A review of paediatric cataract surgery techniques and practices: past, present and future. Niger J Ophthalmol. (2024) 32:2–9. doi: 10.4103/njo.njo_13_23

[62] Wallace RB 3rd. The 45 degree tilt: improvement in surgical ergonomics. J Cataract Refract Surg. (1999) 25:174–6. doi: 10.1016/S0886-3350(99)80122-9, PMID: 9951660

[63] Alberti GN Nose R Schatz M. Do eyelid specula commonly used during cataract surgery affect intraocular pressure? Invest Ophthalmol Vis Sci. (2009) 50:6110.

[64] Mohammadi SF Mazouri A Rahman AN Jabbarvand M Peyman GA. Globe-fixation system for animal eye practice. J Cataract Refract Surg. (2011) 37:4–7. doi: 10.1016/j.jcrs.2010.10.026, PMID: 21183095

[65] Hernaez-Ortega MC Soto-Pedre E. Use of the Honan balloon as a compression device for intravitreal triamcinolone acetonide injection. Ophthalmic Surg Lasers Imaging. (2007) 38:87–8. doi: 10.3928/15428877-20070101-17, PMID: 17278546

[66] Serhan HA Gupta PC Khatib MN Padhi BK Gaidhane S Zahiruddin QS . Effect of intravenous mannitol on intraocular pressure changes in Vitrectomized and non-Vitrectomized eyes: a systematic review and Meta-analysis. Am J Ophthalmol. (2024) 268:45–53. doi: 10.1016/j.ajo.2024.07.013, PMID: 39033830

[67] Huang X Zhou Q Wang S Zhang J Niu G Bi Y. Stepwise decreasing of vitreous pressure by anterior vitrectomy: a novel method for preventing positive vitreous pressure during penetrating Keratoplasty. Adv Ther. (2020) 37:617–29. doi: 10.1007/s12325-019-01139-6, PMID: 31728826

[68] Armarnik S Mimouni M Rosen E Assia EI Segev F. Modified corneal incisions in intraoperative floppy iris syndrome (IFIS)-prone patients. Graefes Arch Clin Exp Ophthalmol. (2016) 254:123–7. doi: 10.1007/s00417-015-3188-7, PMID: 26553196

[69] Al Mahmood AM Al-Swailem SA Behrens A. Clear corneal incision in cataract surgery. Middle East Afr J Ophthalmol. (2014) 21:25–31. doi: 10.4103/0974-9233.124084, PMID: 24669142 PMC3959037

[70] Sharma B Abell RG Arora T Antony T Vajpayee RB. Techniques of anterior capsulotomy in cataract surgery. Indian J Ophthalmol. (2019) 67:450–60. doi: 10.4103/ijo.IJO_1728_18, PMID: 30900573 PMC6446625

[71] Borkenstein AF Borkenstein EM Malyugin B. Ophthalmic Viscosurgical devices (OVDs) in challenging cases: a review. Ophthalmol Ther. (2021) 10:831–43. doi: 10.1007/s40123-021-00403-9, PMID: 34617249 PMC8589875

[72] Oksuz H Daglioglu MC Coskun M Ilhan O Tuzcu EA Ilhan N . Vacuum-assisted continuous circular capsulorhexis using bimanual irrigation and aspiration system of phaco machine in immature cataract. J Ophthalmol. (2013) 2013:921646. doi: 10.1155/2013/921646, PMID: 24303209 PMC3835205

[73] Kothari K Jain SS Shah NJ. Anterior capsular staining with trypan blue for capsulorhexis in mature and hypermature cataracts. A preliminary study. Indian J Ophthalmol. (2001) 49:177–80. PMID: 15887726

[74] Zeng Y Gao JH. Continuous curvilinear Capsulorhexis in cataract surgery using a modified 3-bend Cystotome. J Ophthalmol. (2015) 2015:412810. doi: 10.1155/2015/412810, PMID: 26509078 PMC4609865

[75] Friedman NJ Palanker DV Schuele G Andersen D Marcellino G Seibel BS . Femtosecond laser capsulotomy. J Cataract Refract Surg. (2011) 37:1189–98. doi: 10.1016/j.jcrs.2011.04.022, PMID: 21700099

[76] Grupcheva CN Grupchev DI. CAPSULaser - a new modality in the portfolio of cataract surgeons. Medicine (Baltimore). (2023) 102:e35762. doi: 10.1097/MD.0000000000035762, PMID: 37960729 PMC10637534

[77] Wang Q Jiang ZX Liao RF. Outcomes of 1.8-3.0 mm incision phacoemulsification combined with trabeculectomy for primary angle-closure glaucoma with cataract. Int J Ophthalmol. (2020) 13:246–51. doi: 10.18240/ijo.2020.02.07, PMID: 32090033 PMC7013779

[78] Song Y Zhang Y Li F Zhang Y Lin F Lv A . One-year results of a multicenter study: intraocular pressure-lowering effect of combined phacoemulsification, Goniosynechialysis, and Goniotomy for cases of advanced primary angle-closure Glaucoma with cataract. Asia Pac J Ophthalmol (Phila). (2022) 11:529–35. doi: 10.1097/APO.0000000000000579, PMID: 36417677

[79] Moghimi S Hashemian H Chen R Johari M Mohammadi M Lin SC. Early phacoemulsification in patients with acute primary angle closure. J Curr Ophthalmol. (2015) 27:70–5. doi: 10.1016/j.joco.2015.12.001, PMID: 27239581 PMC4881187

[80] Yu JG Zhao F Xiang Y. Phacoemulsification with Goniosynechialysis versus phacoemulsification alone in angle-closure Glaucoma: a Meta-analysis of randomized controlled trials. J Ophthalmol. (2021) 2021:8831479. doi: 10.1155/2021/8831479, PMID: 33628481 PMC7899770

[81] Chang VS Gibbons A Osigian C. Phacoemulsification in the setting of corneal Endotheliopathies: a review. Int Ophthalmol Clin. (2020) 60:71–89. doi: 10.1097/IIO.0000000000000315, PMID: 32576725 PMC7360340

[82] Upasani D Daigavane S. Phacoemulsification techniques and their effects on corneal endothelial cells and visual acuity: a review of "direct-chop" and "stop-and-chop" approaches under topical anesthesia. Cureus. (2024) 16:e66587. doi: 10.7759/cureus.66587, PMID: 39258086 PMC11385713

[83] Flach AJ. Intraoperative floppy iris syndrome: pathophysiology, prevention, and treatment. Trans Am Ophthalmol Soc. (2009) 107:234–9. PMID: 20126500 PMC2814568

[84] Gothwal VK Wright TA Lamoureux EL Pesudovs K. Measuring outcomes of cataract surgery using the visual function Index-14. J Cataract Refract Surg. (2010) 36:1181–8. doi: 10.1016/j.jcrs.2010.01.029, PMID: 20610098

[85] Sparrow JM Grzeda MT Frost NA Johnston RL Liu CSC Edwards L . Cataract surgery patient-reported outcome measures: a head-to-head comparison of the psychometric performance and patient acceptability of the cat-PROM5 and Catquest-9SF self-report questionnaires. Eye (Lond). (2018) 32:788–95. doi: 10.1038/eye.2017.297, PMID: 29386619 PMC5898871

[86] Hays RD Mangione CM Ellwein L Lindblad AS Spritzer KL McDonnell PJ. Psychometric properties of the National eye Institute-refractive error quality of life instrument. Ophthalmology. (2003) 110:2292–301. doi: 10.1016/j.ophtha.2002.07.001, PMID: 14644710

[87] Hoffer KJ. The Hoffer Q formula: a comparison of theoretic and regression formulas. J Cataract Refract Surg. (1993) 19:700–12. doi: 10.1016/S0886-3350(13)80338-0, PMID: 8271165

[88] Barrett GD. Barrett formulas: strategies to improve IOL power prediction. In: Aramberri J Hoffer KJ Olsen T Savini G Shammas HJ, editors. Intraocular Lens Calculations. Cham: Springer International Publishing (2024). 577–92.

